# Hepatobiliary involvement in Kawasaki disease: from cholestatic hepatitis to the hepatic vascular-biliary unit hypothesis—a state-of-the-art review

**DOI:** 10.3389/fimmu.2026.1852636

**Published:** 2026-08-12

**Authors:** Yong-Xing Zhong, Qi Zheng, Fang-Yan Yang

**Affiliations:** 1 Department of Pediatrics, Shaoxing Maternity and Child Health Care Hospital, Shaoxing, Zhejiang, China; 2 Maternity and Child Health Care Affiliated Hospital, Shaoxing University, Shaoxing, Zhejiang, China; 3 Children’s Hospital, Zhejiang University School of Medicine, Hangzhou, Zhejiang, China

**Keywords:** anakinra, cholestatic hepatitis, hepatic vascular-biliary unit, IVIG resistance, Kawasaki disease, multi-system inflammation, risk stratification

## Abstract

**Background:**

This review provides a comprehensive synthesis of hepatobiliary involvement in Kawasaki disease (KD), with emphasis on cholestatic hepatitis as a clinical sentinel of hyperinflammation. We critically evaluate current evidence, propose an integrated pathogenic framework—the hepatic vascular-biliary unit injury hypothesis—and identify priority research areas.

**Data sources:**

Narrative review of high-quality literature (2015–2025) with inclusion of seminal historical studies, using Oxford Centre for Evidence-Based Medicine (OCEBM) evidence-level grading.

**Results:**

Cholestatic hepatitis occurs in 5.2%–17.8% of acute KD cases, rising to 22%–35% among intravenous immunoglobulin (IVIG)-resistant patients. Large retrospective cohort studies (OCEBM Level 3b) have identified a clinical “risk triangle” comprising IVIG resistance, coronary artery lesions (CALs), and cholestasis as interdependent factors. We propose the hepatic vascular-biliary unit injury hypothesis as an integrated pathogenic framework, supported by correlative pathological and molecular evidence (OCEBM Level 3–4), though causality remains unproven and requires validation in conditional endothelial-specific animal models. Current therapeutic evidence for moderate-to-severe cholestasis derives exclusively from retrospective cohorts and case series (OCEBM Level 3–4). Emerging evidence from Phase I/IIa trials supports the use of interleukin-1 blockade (anakinra) in refractory cases, predominantly derived from studies of coronary artery aneurysms rather than cholestasis-specific populations.

**Conclusions:**

KD-associated cholestatic hepatitis is a critical marker of disease severity with prognostic significance. The hepatic vascular-biliary unit hypothesis provides a mechanistic framework linking systemic vasculitis to cholestasis, pending experimental validation. Definitive evidence for optimal therapeutic strategies is lacking; well-designed randomized controlled trials specifically targeting the cholestasis subpopulation are urgently needed. Given the highest incidence in East Asian populations, clinicians in this region should maintain a particularly high index of suspicion for KD-associated cholestasis, particularly in infants presenting with unexplained jaundice.

## Introduction

Since its initial description by Tomisaku Kawasaki in 1967, our understanding of this acute febrile vasculitis has evolved substantially. Kawasaki disease (KD) remains an enigmatic condition of unknown etiology, yet it has become the predominant cause of acquired heart disease among children in developed nations (1). While coronary artery lesions (CALs) command the greatest clinical attention, extra-cardiac manifestations—once dismissed as benign and self-limited—are now recognized as important reflections of systemic inflammation with independent prognostic value (2).

Among the spectrum of extra-cardiac involvement, hepatobiliary abnormalities represent one of the most frequently encountered complications during acute KD. The clinical presentation spans a continuum from mild, transient transaminitis to overt cholestatic hepatitis, gallbladder hydrops, and, in rare instances, acute liver failure. Cholestatic hepatitis—characterized by conjugated hyperbilirubinemia and elevated serum bile acids—represents the most clinically consequential phenotype, serving as a sentinel marker of systemic hyperinflammation (3).

Epidemiologic data from multicenter cohorts indicate that cholestasis occurs in 5.2%–17.8% of acute KD cases, with markedly higher incidence (22%–35%) among IVIG-resistant patients (4). Importantly, recent retrospective studies have demonstrated that cholestasis is not merely a passive biomarker but an independent risk factor for IVIG resistance, prolonged disease course, and CAL development (OCEBM Level 3b) (5, 6). This triad—IVIG resistance, CALs, and cholestasis—has been proposed as an interdependent “risk triangle” that portends worse outcomes and may reflect a shared underlying pathophysiology of systemic microvascular injury (5, 7). A particularly challenging subset, especially infants < 6 months, may present with cholestatic jaundice as the initial or sole manifestation, substantially delaying diagnosis and optimal IVIG administration (8, 9).

Despite growing clinical awareness, the literature on KD-associated cholestasis remains fragmented. Key gaps include: (1) the absence of an integrated, evidence-based pathogenic framework linking cholestasis to the core immune-vascular pathogenesis of KD; (2) lack of unified diagnostic criteria and prospectively validated severity stratification; (3) insufficient high-level evidence to guide therapeutic decisions, particularly regarding glucocorticoid regimens in IVIG-resistant or severe cases; and (4) limited understanding of the relationship between hepatobiliary involvement and other systemic manifestations such as macrophage activation syndrome (MAS).

This review addresses these gaps through four primary objectives: (i) proposing the hepatic vascular-biliary unit injury hypothesis as an integrated pathogenic framework supported by current correlative evidence, with explicit acknowledgment of its unproven causality; (ii) critically appraising the literature with explicit evidence-level grading; (iii) delineating unresolved controversies and multi-system involvement patterns; and (iv) establishing priority research directions to advance the field.

## Epidemiology and risk factors

### Methods

Databases searched: PubMed/MEDLINE, Web of Science, Scopus, Embase.

Search terms and Boolean combinations: (“Kawasaki disease” OR “mucocutaneous lymph node syndrome”) AND (“cholestasis” OR “cholestatic hepatitis” OR “hepatobiliary” OR “hyperbilirubinemia” OR “bile acid” OR “liver dysfunction” OR “gallbladder hydrops”).

Time range: January 2015 – December 2025, with inclusion of seminal historical studies where foundational.

Date of last search: December 15, 2025.

Inclusion criteria: Peer-reviewed original articles, systematic reviews, meta-analyses, and guidelines; English language; human subjects; reporting hepatobiliary manifestations in acute KD.

Exclusion criteria: Single case reports without unique mechanistic or therapeutic insights; conference abstracts; non-peer-reviewed sources; retracted articles; studies focused exclusively on chronic liver disease unrelated to KD.

This is a narrative review, that OCEBM grading represents author consensus.

#### Incidence and geographic variation

While population-level data on KD-associated cholestasis remain limited, substantial geographic variation has been well-documented for Kawasaki disease itself. A systematic review by Kang et al. covering 34 countries reported the highest KD incidence in East Asian populations (Japan, Korea, Taiwan) compared with European and American cohorts, with incidence rates exceeding 200 per 100,000 children under 5 years in East Asia versus approximately 10–20 per 100,000 in Western countries (4). This geographic gradient likely extends to hepatobiliary manifestations, reflecting shared genetic susceptibility factors including *ITPKC*, *CASP3*, and *ORAI1* polymorphisms that modulate immune hyperactivation (10). Given the elevated incidence in East Asian populations, clinicians practicing in this region should maintain heightened vigilance for KD-associated cholestasis, particularly in infants presenting with unexplained febrile jaundice.

A large retrospective study by Yang et al. (2024) analyzing 1,803 KD patients found that 83 (4.6%) developed cholestasis, with KD being the leading cause of acute febrile cholestasis in children (72.6%) (5). This study identified younger age, hypoalbuminemia, and elevated NT-proBNP as independent risk factors for IVIG unresponsiveness, suggesting that cholestasis may be part of a broader spectrum of systemic capillary leak and endothelial dysfunction (5).

#### Comparison with MIS-C-associated hepatobiliary involvement

Multisystem inflammatory syndrome in children (MIS-C), a post-infectious hyperinflammatory condition associated with SARS-CoV-2, shares substantial clinical overlap with KD, including hepatobiliary manifestations. Elevated transaminases are reported in 40–60% of MIS-C cases, and cholestatic jaundice, though less frequent than in KD, has been documented in severe cases (11, 12). Gallbladder hydrops, once considered relatively specific to KD, has also been observed in MIS-C (12).

Despite these overlaps, important distinctions exist. MIS-C typically presents with more prominent gastrointestinal symptoms (abdominal pain, vomiting, diarrhea) and cardiac dysfunction (myocardial dysfunction, shock), whereas KD predominantly features coronary artery aneurysms. Ferritin and inflammatory markers are generally higher in MIS-C, and the age distribution differs (MIS-C: school-aged children and adolescents; KD: peak under 5 years). Treatment response also varies, with corticosteroids often used as first-line therapy in MIS-C rather than as second-line agents in KD (11, 12).

The shared hepatobiliary involvement across both conditions—arising from distinct triggers but converging on common pathways of endothelial activation and cytokine-mediated injury—strengthens the biological plausibility of the hepatic vascular-biliary unit injury hypothesis proposed in this review. For clinicians, the presence of cholestatic hepatitis or gallbladder hydrops should prompt consideration of both KD and MIS-C, with echocardiography and comprehensive inflammatory marker assessment guiding differential diagnosis and therapy.

#### Age and sex distribution

Age represents the dominant epidemiological determinant. Infants <6 months demonstrate an incidence of 18.9%—approximately twice that of children aged 1–5 years (9.3%)—and present with more severe cholestatic phenotypes, higher rates of IVIG resistance, and more frequent CALs (5, 13). This vulnerability likely reflects immature immune regulatory function, enhanced vascular endothelial susceptibility, and developmental differences in bile acid metabolism (14). Male predominance (male:female = 1.3:1) mirrors the overall sex distribution of KD and may relate to X-linked immune modulators (1).

#### Temporal patterns

Cholestasis predominantly manifests during the acute phase (1–2 weeks after onset), coinciding with peak systemic cytokine elevation. A subset of patients may develop cholestasis during the subacute phase (2–4 weeks), often associated with persistent inflammation, incomplete treatment response, or evolving coronary artery pathology (5, 15). In patients with KD-associated cholestasis, mild cases typically resolve rapidly with effective inflammation control—liver function normalizing within one week after IVIG therapy—whereas those with more severe manifestations, particularly concurrent CALs, may require prolonged follow-up extending beyond 32 months for complete biochemical resolution (3).

#### Independent risk factors and the risk triangle

Robust risk factors for KD-associated cholestasis have been identified in large retrospective cohort studies (OCEBM Level 3b) (3, 5–7) (**Table 1**).

**Table 1:** Risk factors for Kawasaki disease-associated cholestasis.

Risk factor	Odds ratio/relative risk	Evidence level	Proposed mechanism
IVIG resistance	4.2–6.7-fold increased risk	Level 2b	Persistent systemic inflammation and microvascular injury
Elevated CRP, procalcitonin, IL-6, TNF-α	Positive correlation with severity	Level 3a	Cytokine-mediated cholangiocyte transporter dysfunction
Gallbladder hydrops	62.3% co-occurrence	Level 3b	Shared microvascular injury to gallbladder and bile ducts
Hypoalbuminemia	Independent predictor	Level 3b	Systemic capillary leak, hepatic synthetic dysfunction
Hyponatremia	Independent predictor	Level 3b	Syndrome of inappropriate ADH, disease severity marker
Delayed IVIG (>7 days)	Increased risk	Level 3b	Prolonged uncontrolled vascular inflammation
Atypical KD presentation	Increased diagnostic delay	Level 3b	Late treatment initiation, advanced vascular pathology

The Kobayashi scoring system, validated for predicting IVIG non-response, incorporates age, neutrophil percentage, platelet count, aspartate aminotransferase (AST), sodium, C-reactive protein (CRP), and day of illness at treatment initiation (sensitivity 86%, specificity 68%) (16–18). Elevated levels of inflammatory markers including absolute neutrophil count, CRP, procalcitonin, interleukin-6 (IL-6), and tumor necrosis factor-alpha (TNF-α) correlate with disease severity, with concomitant elevations in total bilirubin serving as a specific marker of cholestatic involvement. IVIG-resistant KD patients exhibit significantly higher levels of CRP, neutrophil percentage, and total bilirubin compared with responsive patients. Prediction models incorporating these inflammatory variables have been developed and validated in East Asian populations, demonstrating good predictive performance for identifying patients at risk of IVIG resistance and adverse outcomes (19–21).

#### Pathogenesis: the hepatic vascular-biliary unit injury hypothesis

Prior mechanistic models have attributed KD-associated cholestasis to isolated inflammatory hepatocyte injury or simple bile acid metabolism disorders, lacking integration with the core vascular pathology of KD. Based on recent pathological, molecular, and metabolomic studies, we propose the hepatic vascular-biliary unit injury hypothesis as an integrated pathogenic framework. This hypothesis posits that cholestasis may arise from immune-mediated injury to the hepatic vascular-biliary unit—defined as the functional integration of the peribiliary vascular plexus (the exclusive microvascular supply to cholangiocytes) and cholangiocytes themselves—triggering a cascade of inflammatory amplification, bile acid transporter dysfunction, and intestinal-liver axis disturbance (13, 22).

#### Critical note on evidence strength and hypothesis validation requirements

The evidence supporting this hypothesis is currently correlative (OCEBM Level 3-4), derived from human pathological specimens, immunohistochemistry, and *in vitro* experiments. Causality—whether peribiliary microvascular injury may contribute to cholestasis or whether cholestasis exacerbates microvascular injury—remains unproven. This section critically presents the supporting evidence while explicitly noting limitations, and outlines specific experimental requirements for hypothesis validation.

#### Proposed core initiation: peribiliary vascular plexus endothelial injury

As a systemic medium-vessel vasculitis, KD targets activated endothelial cells, including those of the hepatic artery and its distal peribiliary vascular plexus (23, 24). This microvascular network constitutes the sole oxygenated blood supply to cholangiocytes, rendering them uniquely vulnerable to ischemic injury during systemic vasculitis.

Inflammatory cell infiltration, endothelial swelling, and microthrombosis within the peribiliary vascular plexus have been documented in liver biopsies from children with KD-associated cholestasis (25). Downregulation of vascular endothelial growth factor (VEGF) and endothelial nitric oxide synthase (eNOS) in hepatic vascular endothelial cells suggests impaired endothelial repair capacity and persistent vasogenic dysfunction (22). Comparative studies of other systemic vasculitides with hepatobiliary involvement (e.g., IgA vasculitis-associated cholangiopathy) support the concept of microvascular injury as a shared pathogenic mechanism (23). While the liver biopsy findings described above provide compelling correlative evidence supporting the hepatic vascular-biliary unit injury hypothesis, several important limitations must be acknowledged to properly contextualize their contribution. First, the biopsy data are derived from fewer than ten published cases, all of which represent extreme clinical phenotypes characterized by severe cholestasis, IVIG resistance, or diagnostic uncertainty warranting tissue sampling. This introduces unavoidable selection bias, as patients undergoing liver biopsy constitute the most severely affected subset of KD-associated cholestasis and may not reflect the full clinical spectrum of this complication. Second, the generalizability of these pathological observations to patients with mild-to-moderate cholestasis—who comprise the majority of KD-associated hepatobiliary involvement—remains uncertain, as biopsy is not indicated in such cases. Third, liver biopsy in the acute phase of KD is ethically constrained due to the risks of bleeding in a systemically inflamed, potentially coagulopathic patient, as well as the availability of non-invasive alternatives, further limiting the evidence base. These limitations underscore the need for validation of the pathological findings in animal models that recapitulate KD vasculitis and, where feasible, through less invasive mechanistic studies such as transcriptomic or proteomic profiling of peripheral blood or bile-acid-based metabolomic signatures that may serve as surrogate markers of the proposed microvascular-biliary injury pathway (22, 25).

##### Critical limitation and validation requirements

These findings establish a spatial and temporal association between peribiliary microvascular injury and cholestasis. However, they do not prove causation. Definitive validation requires: (i) animal models recapitulating KD vasculitis with conditional endothelial-specific gene targeting (e.g., VE-cadherin-Cre driven) to determine whether isolated microvascular injury is sufficient to induce cholestasis independent of systemic inflammation; (ii) longitudinal studies establishing temporal precedence of microvascular changes relative to biochemical cholestasis; and (iii) interventional studies demonstrating that microvascular protection prevents or ameliorates cholestasis.

#### Proposed inflammatory amplification: cytokine-mediated cholangiocyte injury

Acute KD is characterized by a cytokine storm dominated by IL-1β, IL-6, TNF-α, and interferon-γ (IFN-γ) (19, 26). Experimental mouse models have established the critical role of IL-1β in KD vasculitis pathogenesis, with IL-1 receptor blockade showing therapeutic efficacy—providing mechanistic rationale for biologic therapy in refractory cholestatic cases (26).

##### Supporting evidence

*In vitro* studies demonstrate that TNF-α and IL-6 activate nuclear factor-κB (NF-κB) signaling in cholangiocytes, downregulating mRNA and protein expression of key bile acid transporters: bile salt export pump (BSEP/ABCB11), multidrug resistance-associated protein 2 (MRP2/ABCC2), and sodium taurocholate cotransporting polypeptide (NTCP/SLC10A1) (27, 28).

BSEP and MRP2 dysfunction may lead to intracellular toxic bile acid accumulation, inducing cholangiocyte apoptosis, portal inflammation, and secondary biliary structural injury (29, 30).

Elevated inflammatory markers in cholestatic KD patients correlate with transporter downregulation, supporting a clinically relevant cytokine-cholangiocyte axis (22).

##### Critical limitation and validation requirements

*In vitro* findings require validation *in vivo* using cholangiocyte-specific reporter models. Whether cholangiocyte transporter dysfunction in KD patients directly results from cytokine exposure versus secondary effects of microvascular ischemia (or both, in a feed-forward loop) remains unresolved. Required studies include: (i) single-cell RNA sequencing of cholangiocytes from KD liver biopsies; (ii) organoid models incorporating both vascular and biliary components; and (iii) studies of cytokine blockade effects on liver biochemistry independent of systemic inflammation control.

#### Proposed secondary disorder: bile acid metabolism and intestinal-liver axis disturbance

Targeted metabolomic studies have revealed significant alterations in bile acid profiles among KD patients with cholestasis: elevated serum total bile acids, increased toxic unconjugated bile acids (chenodeoxycholic acid, lithocholic acid), and decreased conjugated-to-unconjugated bile acid ratio (22). Toxic bile acid accumulation further damages hepatocytes and cholangiocytes via farnesoid X receptor (FXR)-mediated and bile acid receptor (TGR5)-independent pathways, establishing a secondary amplification loop.

Additionally, acute KD is associated with intestinal dysbiosis (reduced *Bifidobacterium* and *Lactobacillus*, increased *Klebsiella* and *Enterobacter*), increased mucosal permeability, and endotoxin translocation, disrupting the intestinal-liver axis, activating hepatic Kupffer cells via toll-like receptor 4 (TLR4), and amplifying local inflammation—potentially perpetuating cholestasis even after systemic inflammation is controlled (31). Recent single-cell RNA sequencing studies have identified enhanced bile acid metabolism pathways in monocytes and NK cells from KD patients, with *VIM*, *CLIC1*, and *ITGB2* emerging as novel metabolism-related biomarkers potentially linking immune cell activation to hepatobiliary injury (32).

##### Critical limitation and validation requirements

The temporal relationship between bile acid alterations and cholestasis onset has not been established; current metabolomic profiles are cross-sectional and cannot distinguish cause from consequence. Prospective serial sampling from disease onset through convalescence is required to define the natural history of bile acid perturbations and their relationship to clinical cholestasis.

#### Multi-system involvement: cholestasis as part of a hyperinflammatory spectrum

KD-associated cholestasis frequently co-occurs with other severe systemic manifestations, suggesting shared pathogenic mechanisms:

MAS: Occurring in 1.1–1.8% of KD cases, MAS represents the extreme end of the hyperinflammatory spectrum and shares with severe cholestasis the features of persistent fever, cytopenias, hyperferritinemia, and coagulopathy (30). The shared pathogenic drivers—uncontrolled T-cell and macrophage activation, elevated IL-1β and IL-6, and systemic endothelial injury—suggest that severe cholestatic KD and MAS may exist on a pathogenic continuum. Differentiating the two is critical: ferritin >5,000 ng/mL, progressive thrombocytopenia (platelets <150 × 10^9^/L), and fibrinogen <1.5 g/L favor MAS, whereas the presence of thrombocytosis and ferritin in the 1,000--5,000 ng/mL range is more consistent with severe cholestasis without overt MAS. Notably, current HLH-2004 diagnostic criteria include transaminases but not cholestatic parameters; we propose that incorporating direct bilirubin and serum bile acids may improve screening sensitivity in KD-associated MAS, though this requires prospective validation and should be interpreted with clinical judgment. Clinical vigilance for MAS is essential in any KD patient with cholestatic hepatitis who develops new cytopenias, rising ferritin, or unexplained coagulopathy, as early recognition and prompt, guideline-directed immunosuppression (glucocorticoids and/or anakinra) is generally recommended by experts and may be life-saving in this severe context, although the evidence base for this specific recommendation remains predominantly derived from case series and expert opinion (OCEBM Level 4–5).

Coronary Artery Lesions: The “risk triangle” of IVIG resistance, CALs, and cholestasis implies that these manifestations may represent differential organ vulnerability to a shared systemic microvasculopathy.

Meningoencephalitis: Central nervous system involvement and cholestasis may both reflect systemic microvascular dysfunction with organ-specific susceptibility.

**Figure 1:**
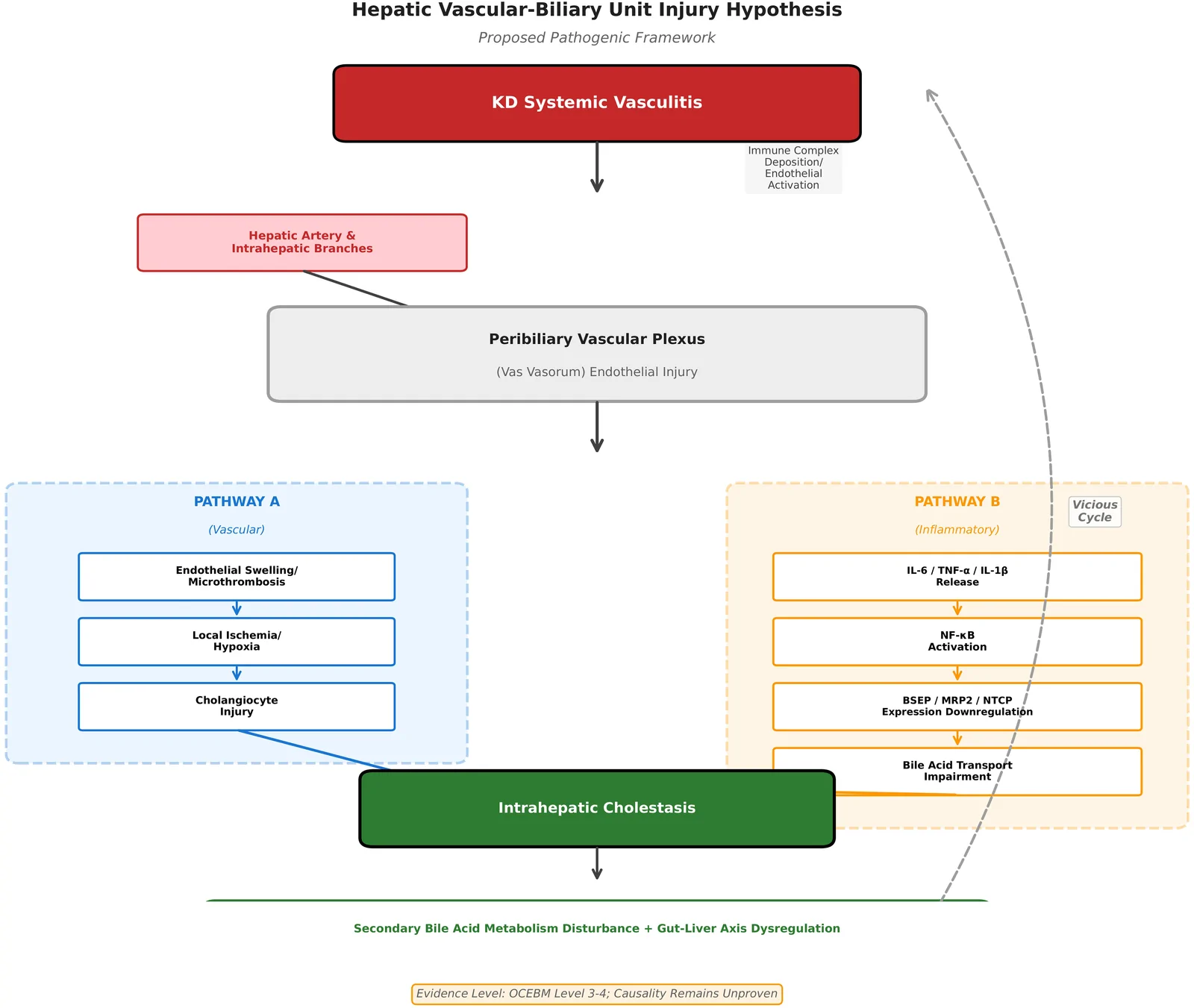
The hepatic vascular-biliary unit injury hypothesis in Kawasaki disease-associated cholestasis. This schematic represents a hypothesis, not an established pathogenic mechanism. All depicted pathways are supported by correlative evidence only (OCEBM Level 3–4). Causality remains unproven. **(A)** Acute KD triggers systemic vasculitis with endothelial activation and a cytokine storm (IL-1β, IL-6, TNF-α, IFN-γ). **(B)** The peribiliary vascular plexus—the exclusive microvascular supply to cholangiocytes—undergoes endothelial injury, swelling, and microthrombosis. **(C)** Two synergistic pathways may lead to cholangiocyte dysfunction: Pathway 1 (ischemic) involves microvascular occlusion with VEGF/eNOS downregulation, compromising cholangiocyte oxygenation; Pathway 2 (cytokine-mediated) involves NF-κB-dependent transcriptional repression of bile acid transporters (BSEP, MRP2, NTCP), causing intracellular toxic bile acid retention. **(D)** Established cholestasis shifts the bile acid pool toward toxic unconjugated species and disrupts the gut-liver axis (dysbiosis, endotoxin translocation, Kupffer cell activation), creating a self-perpetuating amplification loop (dashed arrow). This integrated pathogenic framework is synthesized from the published evidence reviewed in this article. Evidence level: OCEBM Level 3-4 (correlative). Causality remains unproven; definitive validation requires conditional endothelial- and cholangiocyte-specific animal models recapitulating KD vasculitis. BSEP, bile salt export pump (ABCB11); eNOS, endothelial nitric oxide synthase; IFN-γ, interferon-gamma; IL, interleukin; KD, Kawasaki disease; MRP2, multidrug resistance-associated protein 2 (ABCC2); NF-κB, nuclear factor-kappa B; NTCP, sodium taurocholate cotransporting polypeptide (SLC10A1); TNF-α, tumor necrosis factor-alpha; VEGF, vascular endothelial growth factor.

This multi-system pattern supports the conceptualization of cholestasis not as an isolated hepatobiliary complication, but as a marker of severe systemic vascular-biliary unit pathology requiring comprehensive monitoring and aggressive anti-inflammatory intervention. A critical appraisal of the hepatic vascular-biliary unit hypothesis reveals a landscape of correlative support juxtaposed with substantial knowledge gaps. Supportive evidence includes: (i) histopathological documentation of peribiliary microvascular inflammation, endothelial swelling, and microthrombosis in liver biopsies from severe KD cholestasis cases (though derived from fewer than ten published extreme phenotypes); (ii) *in vitro* data showing that TNF-α and IL-6 downregulate cholangiocyte bile acid transporters (BSEP, MRP2, NTCP) via NF-κB activation; and (iii) metabolomic profiling demonstrating altered bile acid pools and gut–liver axis disruption in acute KD. However, conflicting or absent evidence is equally notable: the biopsy findings are subject to selection bias and lack generalizability to mild-to-moderate cases; the *in vitro* transporter studies await *in vivo* confirmation in cholangiocyte-specific models; and metabolomic data are cross-sectional, precluding any temporal inference of causation. Three fundamental unanswered questions persist: (i) whether peribiliary microvascular injury initiates cholestasis or instead represents a secondary consequence of bile acid toxicity—or both in a self-perpetuating loop; (ii) whether elevated circulating bile acids actively contribute to coronary artery endothelial dysfunction beyond the hepatobiliary compartment; and (iii) which genetic or immunological modifiers determine organ-specific vulnerability. Definitive validation of the hypothesis requires conditional endothelial-specific (VE-cadherin-Cre) and cholangiocyte-specific (KRT19-Cre) mouse models that recapitulate KD vasculitis, longitudinal studies establishing the temporal precedence of microvascular changes over biochemical cholestasis, and interventional experiments demonstrating that microvascular protection or targeted cytokine blockade ameliorates cholestasis independent of systemic inflammation control. Until such evidence emerges, the hypothesis should be regarded as a mechanistically plausible but unproven framework to guide future investigation, rather than an established pathogenic paradigm. As illustrated in **Figure 1**, this proposed pathogenic framework is synthesized from the published evidence reviewed in this article.

## Clinical, laboratory, and imaging features

### Clinical manifestations

KD-associated cholestasis lacks pathognomonic symptoms, requiring high clinical suspicion. Two phenotypic patterns are recognized (3, 5) (**Table 2**).

**Table 2:** Clinical phenotypes of Kawasaki disease-associated cholestasis.

Phenotype	Characteristics	Clinical implication
Typical	Cholestatic symptoms 3–7 days after KD onset: scleral icterus, acholic stools, dark urine, abdominal distension, anorexia, right upper quadrant tenderness; gallbladder hydrops in 30%–50%	Diagnosis typically follows KD diagnosis; echocardiography essential
Atypical/Isolated	Isolated persistent fever + cholestatic jaundice as initial/only manifestation; lacking classic KD criteria (conjunctival injection, oral changes, extremity changes, rash, cervical adenopathy); predominantly infants <6 months	High misdiagnosis rate (viral hepatitis, biliary atresia, sepsis); echocardiography is essential and may be diagnostic

Diagnostic Alert: For children < 5 years with unexplained fever >5 days and cholestatic jaundice, KD must be prioritized in differential diagnosis, with prompt echocardiography to screen for CALs even in the absence of other classic features (5).

Several case reports have documented KD with cholestatic hepatitis as the presenting feature, including cases with concomitant *Mycoplasma pneumoniae* infection (33) and adolescent-onset disease with hepatic predominance (16). Acute cholestasis as an uncommon onset of KD has been increasingly recognized (34).

### Laboratory characteristics

Laboratory findings reflect combined obstructive cholestasis and systemic inflammation (**Table 3**).

**Table 3:** Laboratory characteristics of Kawasaki disease-associated cholestasis.

Category	Key findings	Interpretation
Cholestasis markers	Direct bilirubin >17.1 μmol/L (or >50% of total); serum bile acids >10 μmol/L (more sensitive early marker than bilirubin); elevated ALP, γ-GT; mild-to-moderate ALT/AST elevation	Biliary pattern with mixed hepatocellular injury; bile acids may elevate before clinical jaundice
Inflammatory markers	Elevated CRP, ESR, procalcitonin; neutrophilia; hypoalbuminemia; hyponatremia; reactive thrombocytosis (subacute phase)	Reflects systemic cytokine storm and capillary leak
Severe cases	Mild coagulation dysfunction (prolonged PT, low fibrinogen); hyperferritinemia (suspect MAS); acute liver failure is extremely rare	Indicates advanced disease with synthetic dysfunction; consider MAS workup

A comprehensive review by Suhaini et al. detailed the spectrum of hepatobiliary manifestations in KD, emphasizing that liver involvement ranges from asymptomatic transaminitis to severe cholestasis requiring intensive intervention (25).

### Imaging features

Abdominal ultrasound is the first-line imaging modality (non-invasive, no radiation, high feasibility, bedside availability). Gallbladder hydrops is highly characteristic of KD, defined as an enlarged, thin-walled, tense gallbladder without stones or mechanical obstruction (34). Additional findings include mild intrahepatic bile duct dilatation suggesting functional biliary stasis, hepatomegaly, and increased parenchymal echogenicity representing non-specific inflammatory edema (35).

For persistent cholestasis exceeding four weeks, magnetic resonance cholangiopancreatography (MRCP) is recommended to exclude congenital biliary anomalies (biliary atresia, choledochal cyst) or organic obstruction, consistent with standard pediatric cholestasis diagnostic algorithms and supported by case reports demonstrating the utility of MRI in evaluating hepatobiliary involvement in Kawasaki disease (36).

## Diagnosis, differential diagnosis, and severity stratification

A comparative summary of key differential diagnostic considerations, including clinical, laboratory, and imaging discriminators, is presented in **Table 4**.

**Table 4:** Differential diagnosis.

Disease	Fever pattern	Key laboratory	Imaging	Key discriminator
KD cholestasis	Persistent >5 days	Obstructive pattern; markedly elevated CRP/ESR; thrombocytosis (subacute)	Gallbladder hydrops; no organic obstruction; ± CALs on echo	Fever precedes/coincides with jaundice; presence of CALs on echocardiography is highly suggestive (but absence does not exclude)
MIS-C	Persistent or relapsing	Elevated CRP, ESR, ferritin; lymphopenia; elevated troponin/BNP	± Gallbladder hydrops; ± myocardial dysfunction; ± coronary dilation	SARS-CoV-2 exposure/serology positive; prominent GI symptoms (abdominal pain, vomiting, diarrhea) and cardiac involvement; older age (school-aged children) than typical KD
EBV hepatitis	Febrile (acute)	Hepatocellular pattern (ALT/AST > ALP); atypical lymphocytosis; thrombocytopenia possible	Non-specific; ± hepatosplenomegaly	VCA-IgM^+^; EBV-DNA^+^; tonsillitis/pharyngitis prominent; heterophile antibody positive (in older children)
CMV hepatitis	Variable, often low-grade	Hepatocellular pattern; mild transaminitis	Non-specific	CMV-IgM^+^; CMV-DNA^+^; often milder clinical course; may have prolonged fever but lacks KD mucocutaneous features
Adenovirus hepatitis	High fever, prolonged	Hepatocellular or mixed pattern; markedly elevated transaminases	Non-specific; ± hepatomegaly	Adenovirus PCR^+^ (respiratory or stool); rapid progression in immunocompromised; may cause fever >5 days but lacks conjunctival injection and extremity changes
Sepsis-associated cholestasis	Persistent, often with hemodynamic instability	Elevated procalcitonin; positive cultures; multi-organ dysfunction	Non-specific; ± hepatomegaly	Evidence of bacterial/fungal infection; responds to antimicrobial therapy; no KD-specific features
Viral hepatitis (HAV/HBV/HCV)	Febrile (acute)	Hepatocellular pattern (ALT/AST > ALP); viral serology positive; normal platelets	No hydrops; ± hepatomegaly	Jaundice precedes/fever follows; specific IgM positive; epidemiologic context
Biliary atresia	Afebrile	Obstructive pattern; ALP/γ-GT > ALT/AST; normal inflammatory markers	Biliary stricture; contracted/absent gallbladder; triangular cord sign	Acholic stools from birth; onset <3 months; no response to IVIG
Drug-induced liver injury	± Fever	Mixed pattern; eosinophilia may be present	Non-specific	Temporal relationship to drug initiation; resolution with withdrawal; rechallenge positive
Autoimmune hepatitis	± Low-grade fever	Hepatocellular or mixed; autoantibodies (+); elevated IgG; interface hepatitis on histology	Non-specific	Chronic presentation; female predominance; response to immunosuppression

### Proposed diagnostic framework (expert consensus, OCEBM Level 5)

No unified international diagnostic criteria currently exist. Based on published expert consensus (2015-2025), we propose the following framework pending prospective validation (**Table 5**).

**Table 5:** Severity stratification (expert consensus, OCEBM level 5; pending prospective validation).

Severity	Direct bilirubin	Serum bile acids	Gallbladder hydrops	IVIG resistance	CALs	Proposed management
Mild	17.1–34.2 μmol/L	Normal or mildly elevated (<20 μmol/L)	Absent	Absent	Absent	IVIG monotherapy + supportive care
Moderate	34.2–85.5 μmol/L	Elevated (20–100 μmol/L)	Mild/moderate	Absent	Absent	IVIG + early glucocorticoid consideration
Severe	>85.5 μmol/L	Markedly elevated (>100 μmol/L)	Large/prolonged	Present or high risk	Present or high risk	IVIG + combination glucocorticoid; consider biologic therapy

1. Confirmed KD (typical or atypical, per AHA 2017 criteria) (1).
2. Cholestasis onset during acute or subacute phase (≤4 weeks from disease onset).
3. Laboratory confirmation: Direct bilirubin >17.1 μmol/L or serum bile acids >10 μmol/L with elevated ALP/γ-GT.
4. Exclusion of alternative cholestatic etiologies (viral hepatitis, congenital biliary malformation, drug-induced liver injury, metabolic disease, sepsis-associated cholestasis).

The diagnostic approach aligns with the joint NASPGHAN/ESPGHAN guidelines for the evaluation of cholestatic jaundice in infants (37).

**Figure 2:**
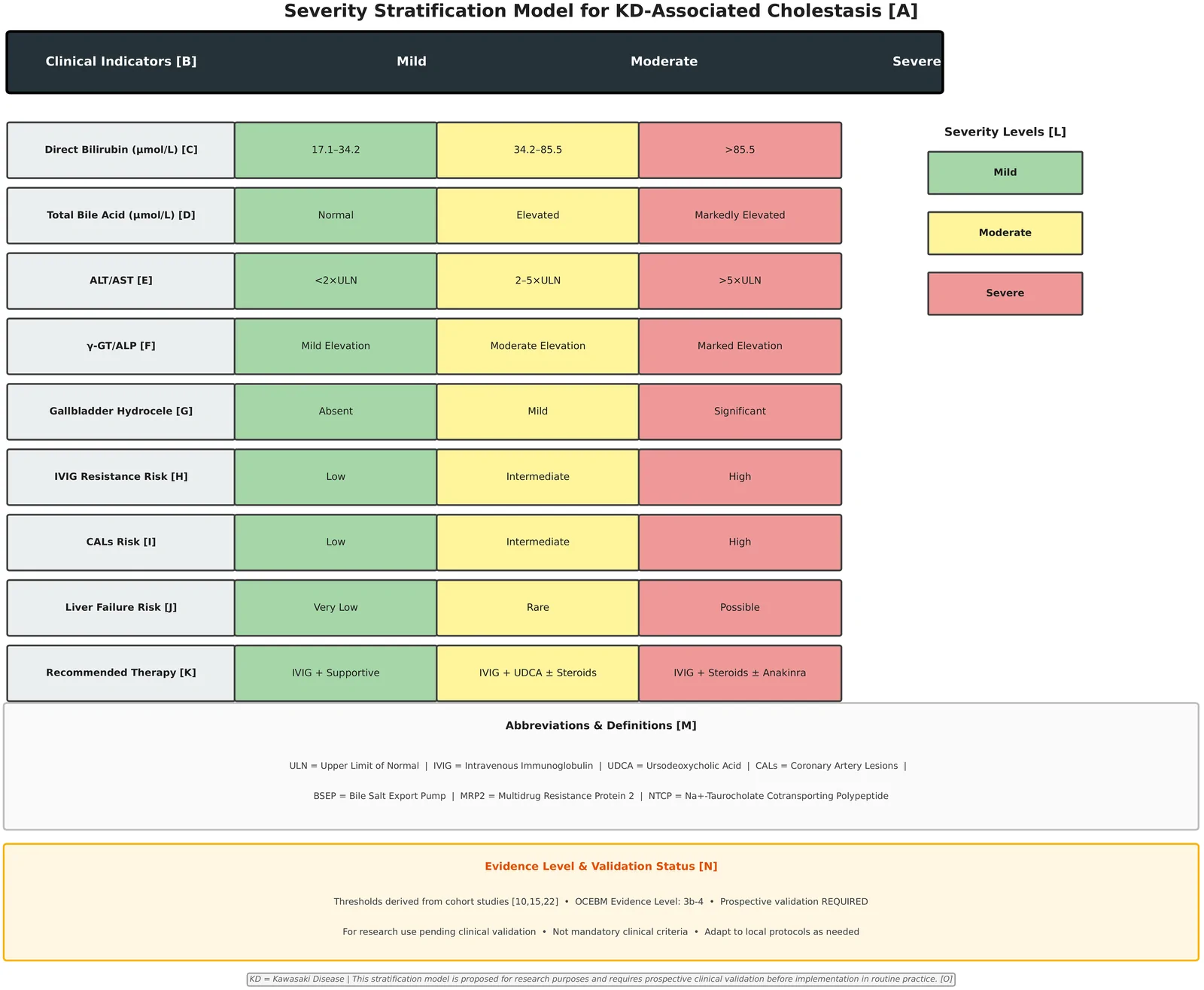
Proposed severity stratification model for KD-associated cholestasis (hypothesis-generating, awaiting prospective validation). Proposed three-tier classification based on retrospective cohort data (OCEBM Level 3b) and expert consensus. Thresholds require prospective validation, particularly in high-incidence East Asian populations. Thresholds have not been prospectively validated and should be used solely for research purposes, not for clinical decision-making. The recommended management strategies represent expert suggestions and are classified as [Expert Hypothesis · Grade D] unless otherwise supported by higher-level evidence **Table 6**.

Threshold Derivation: Cutoff values derived from retrospective cohort studies (5, 13, 22, 38, 39) and expert consensus. These thresholds require prospective validation in diverse populations, particularly across Asian countries where KD incidence is highest and genetic backgrounds may influence severity phenotypes. A visual summary of this proposed severity stratification model is presented in **Figure 2**.

**Table 6:** Proposed severity stratification model for KD-associated cholestasis.

Severity	Direct bilirubin	Serum bile acids	Gallbladder hydrops	IVIG resistance risk	CAL risk	Recommended management
Mild	17.1 - 34.2 μmol/L	<20 μmol/L	Absent	Low	Low	IVIG monotherapy
Moderate	34.2 - 85.5 μmol/L	20 - 100 μmol/L	Mild/transient	Intermediate	Low	IVIG + strongly consider early UDCA ± early glucocorticoid
Severe	>85.5 μmol/L	>100 μmol/L	Large/persistent	High/Present	High/Present	Initial combination therapy preferred + UDCA; consider anakinra if refractory

CALs, coronary artery lesions; IVIG, intravenous immunoglobulin; MAS, macrophage activation syndrome; UDCA, ursodeoxycholic acid.

## Therapeutic landscape: current evidence and critical gaps

Treatment of KD-associated cholestasis is based on standard KD anti-inflammatory therapy combined with supportive hepatoprotective measures. The central clinical dilemma—choice between IVIG-glucocorticoid sequential versus combination therapy for moderate-to-severe cases—is currently unsupported by high-level evidence specific to the cholestasis subpopulation. This section critically appraises available data, explicitly noting when evidence is derived from general KD or coronary artery aneurysm populations rather than cholestasis-specific studies.

### Standard first-line therapy

Single-dose IVIG (2 g/kg) remains first-line for all KD, controlling systemic inflammation and resolving mild cholestasis within 3–7 days in IVIG-sensitive patients (1, 13). Most mild cases require no additional hepatoprotective therapy beyond supportive care.

### Supportive hepatoprotective therapy

Critical Evidence Gap: No RCTs have evaluated whether early UDCA initiation (within 48 hours of diagnosis) improves cholestasis resolution, reduces IVIG resistance, or prevents CALs compared with delayed initiation after systemic inflammation control. This represents a high-priority research question (**Table 7**).

**Table 7:** Supportive hepatoprotective therapy.

Agent	Indication	Evidence level	Key evidence gap	Notes
Ursodeoxycholic acid (UDCA)	Moderate-to-severe cholestasis	Level 3b-4	No RCT evaluating early vs. delayed initiation; optimal duration undefined	10–20 mg/kg/day; promotes bile flow; protects cholangiocytes from toxic bile acids; generally well-tolerated
Adenosylmethionine/reduced glutathione	Adjunctive hepatocyte protection	Level 5	No pediatric-specific efficacy data	Limited evidence in KD; theoretical antioxidant benefit
Albumin infusion	Severe hypoalbuminemia (<2.5 g/dL)	Level 5	No RCT demonstrating outcome benefit	Maintains colloid osmotic pressure; may bind unconjugated bilirubin

### Glucocorticoid regimens: sequential vs. combination therapy

Glucocorticoids are indicated as second-line for IVIG-resistant or severe KD-associated cholestasis. Two regimens are used clinically; no head-to-head RCTs exist in cholestasis-specific populations (OCEBM Level 3b-4) (40, 41) (**Table 8**).

**Table 8:** Glucocorticoid regimens: sequential vs. combination therapy.

Regimen	Protocol	Advantages	Disadvantages	Evidence base
Sequential	IVIG (2 g/kg) → evaluate response at 36–48h → if resistant or severe cholestasis persists, add methylprednisolone (1–2 mg/kg/d) or prednisone (1–2 mg/kg/d), taper over 2–4 weeks	Avoids steroid exposure in IVIG-sensitive patients; theoretically lower steroid-related adverse effects	Delayed anti-inflammatory effect in severe cases; potentially longer cholestasis duration; possibly higher CAL risk in severe subset with delayed control	Retrospective cohorts in general KD; no cholestasis-specific RCTs
Combination	IVIG + methylprednisolone (30 mg/kg/d for 3 days) or prednisone (2 mg/kg/d) initiated concurrently	Early synergistic anti-inflammatory effect; faster cholestasis resolution (biochemical improvement observed)	Higher transient steroid-related adverse effects (hyperglycemia, hypertension); uncertain long-term safety	RAISE trial [45] and subsequent studies in severe KD; no cholestasis-specific RCTs

Implication for Cholestasis Management: Given the direct relationship between cholestasis severity and systemic inflammation, and the potential for irreversible biliary injury with delayed control, combination therapy may be considered for patients with severe cholestasis (direct bilirubin >85.5 μmol/L) even in the absence of confirmed IVIG resistance, recognizing that this recommendation is based on mechanistic rationale and expert consensus (OCEBM Level 5) rather than direct clinical trial evidence [Level 2b, extrapolated from severe KD; no cholestasis-specific RCTs; Expert Hypothesis · Grade D].

### Emerging biologic therapies: evidence and caveats

#### Interleukin-1 blockade (anakinra)

Anakinra has emerged as the most promising biologic for refractory KD. Critical caveat: The evidence base derives predominantly from studies of IVIG-resistant KD or coronary artery aneurysms, not specifically from cholestasis-defined populations. No cholestasis-specific data exist for anakinra. Its inclusion here is based solely on mechanistic rationale (IL-1β blockade) and extrapolation from coronary artery aneurysm populations. Its efficacy for cholestasis resolution remains undefined.

No cholestasis-specific data exist for anakinra. Its inclusion here is based solely on mechanistic rationale (IL-1β blockade) and extrapolation from coronary artery aneurysm populations. Its efficacy for cholestasis resolution remains undefined.

Implication for Cholestasis Management:

While anakinra demonstrates clear efficacy in refractory systemic KD inflammation, its specific efficacy for cholestasis resolution—versus general inflammation control—has not been isolated. Given the shared IL-1β-driven pathogenesis, anakinra is rational for refractory cholestatic cases, but cholestasis-specific response rates remain undefined (**Table 9**).

**Table 9:** Evidence base for anakinra in refractory Kawasaki disease.

Study	Design	Population	Key findings	Limitations
Kone-Paut et al., 2018 (42)	Retrospective case series	11 IVIG-resistant KD	Rapid fever resolution (24-48h), significant CRP reduction	No cholestasis stratification; no control group
Yang et al., 2022 (43)	Phase I/IIa trial	22 KD with coronary artery aneurysms	Adequate tolerability; significant reduction in IL-6, IL-8, TNF-α; 82% CAL regression at 6 weeks	Primary endpoint was CAL regression, not cholestasis; small sample
Gambacorta et al., 2020 (29)	Case report	1 infant with refractory KD, giant CAA	Complete CAA resolution	Single case; no cholestasis focus
Bossi et al., 2022 (30)	Case report	1 infant with recurrent KD + MAS	Efficacy in MAS complication	Single case; complex presentation
Kone-Paut et al., 2021 (42)	Phase II open-label	IVIG-resistant KD	75% fever resolution; significant CRP reduction	No control group; heterogeneous population
Tremoulet et al., 2024 (44)	Phase 1/2a trial	Refractory KD	Confirmed safety profile; no serious adverse events	Safety-focused; efficacy data preliminary
Ferrara et al., 2020 (45)	Systematic review	Treatment-resistant KD	Concluded effective and safe; called for controlled trials	Heterogeneous studies; no cholestasis-specific analysis
Inguscio et al., 2025 (46)	Multicenter case series	Infants <6 months with KD + CALs	Favorable outcomes in high-risk population	Retrospective; CAL-focused, not cholestasis-specific

All data are derived from general IVIG-resistant KD or coronary artery aneurysm populations; no cholestasis-specific data exist. Evidence level: OCEBM Level 2b–3b.

### TNF-α inhibition (infliximab)

Given the central pathogenic role of TNF-α within the hepatic vascular-biliary unit injury hypothesis, TNF-α blockade represents a mechanistically rational intervention for refractory KD-associated cholestasis. Infliximab, a chimeric monoclonal antibody against TNF-α, is an established second- or third-line agent for IVIG-resistant KD (1). Case reports have documented clinical and biochemical improvement following infliximab administration in children with KD complicated by severe cholestatic hepatitis (OCEBM Level 4) (e.g., PMID: 28947445, 23190890). However, these observations are limited to isolated cases, and no studies have specifically evaluated infliximab efficacy in a cholestasis-defined cohort. Furthermore, clinicians must remain cognizant of the rare but recognized risk of infliximab-induced liver injury—ranging from transient transaminase elevation to autoimmune-like hepatitis—in patients with pre-existing hepatic dysfunction. Therefore, while TNF-α inhibition is theoretically sound, it should be reserved for scenarios where IL-1 or IL-6 blockade is unavailable or contraindicated, pending the generation of cholestasis-specific evidence.

### Other biologic and salvage options

Beyond the standard and glucocorticoid-based regimens discussed above, several other biologic and salvage modalities have been explored in refractory or life-threatening KD-associated cholestasis, though the evidence base remains limited to small case series and expert experience. A summary of the comparative effectiveness, mechanisms of action, and key limitations of these alternative therapeutic options is provided in **Table 10**.

**Table 10:** Other biologic and salvage options.

Therapy	Mechanism	Evidence base	Population	Efficacy in cholestasis
Tocilizumab	Anti-IL-6 receptor mAb	Case series (n<20)	Refractory KD, some with MAS	70–80% response in refractory systemic inflammation; cholestasis-specific data absent
Plasma exchange	Removal of inflammatory mediators, cytokine storm control	Case reports	Hyperbilirubinemia >170 μmol/L, coagulopathy, or MAS	Reserved for life-threatening complications; mechanical clearance of bile acids unproven

### Summary of therapeutic evidence

Given the heterogeneity of study designs, patient populations, and outcome measures across the currently available literature, synthesizing the therapeutic evidence for KD-associated cholestasis poses a considerable challenge. To facilitate clinical reasoning and highlight critical knowledge gaps, we have compiled a comprehensive summary of the available evidence for each major therapeutic approach, with explicit OCEBM level grading, recommendation strength, and specific evidence limitations, as presented in **Table 11**.

**Table 11:** Summary of therapeutic evidence.

Therapy	Indication	OCEBM level	Recommendation strength	Evidence limitation
IVIG monotherapy	Mild cholestasis	Level 2b	Strong	No RCT specifically for cholestasis subset; extrapolated from general KD
Sequential IVIG-glucocorticoid	Moderate cholestasis, IVIG-responsive	Level 3b	Weak	Retrospective cohorts only; no cholestasis-specific RCTs
Combination IVIG-glucocorticoid	Severe cholestasis, IVIG resistance, CALs	Level 2b	Weak to Moderate	RAISE trial in severe KD, not cholestasis-specific; mechanistically rational
Anakinra	Refractory severe cholestasis, CALs, MAS	Level 2b-3b	Weak to Moderate (emerging)	Phase I/IIa trials, small samples; evidence derived from CAL/MAS populations, not cholestasis-specific
Infliximab	Refractory severe, alternative to IL-1 blockade	Level 4	Very weak	Case reports only; no cholestasis-specific data; monitor for hepatotoxicity
Tocilizumab	Refractory severe	Level 4	Very weak	Case series, n<20; no cholestasis focus
Plasma exchange	Hyperbilirubinemia >170 μmol/L, coagulopathy	Level 5	Very weak	Case reports; reserved for emergency rescue

**Figure 3:**
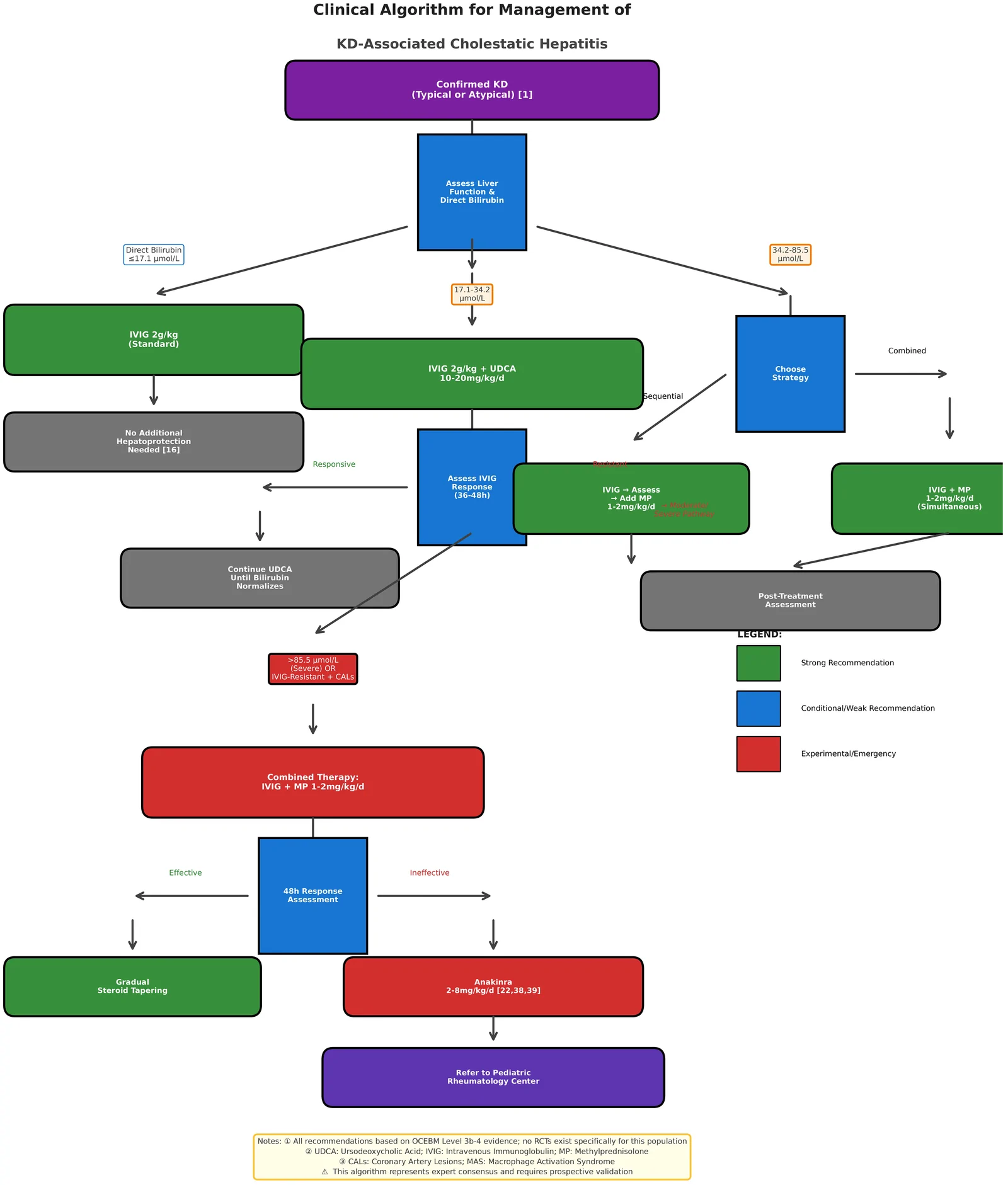
Clinical algorithm for KD-associated cholestatic hepatitis. Proposed direct bilirubin thresholds (mild 17.1-34.2 μmol/L; moderate 34.2-85.5 μmol/L; severe >85.5 μmol/L) require prospective validation. Algorithm integrates OCEBM Level 2b-4 evidence with expert consensus. This algorithm is a hypothesis-generating framework, not validated clinical guidance. All glucocorticoid, UDCA, and biologic therapy recommendations within this algorithm are classified as Expert Hypothesis-Grade D unless otherwise referenced to higher-level evidence. This algorithm should not be used to guide clinical practice outside of approved research protocols. Step 1: Diagnosis. Confirm KD (AHA 2017 criteria), biochemical cholestasis (direct bilirubin >17.1 μmol/L or bile acids >10 μmol/L), and exclude alternative etiologies. Abdominal ultrasound and echocardiography are mandatory—CAL screening is essential even with incomplete KD criteria. Step 2: Stratification. Apply proposed severity criteria (see **Figure 2**). Step 3: Risk assessment. Evaluate IVIG resistance risk (Kobayashi score, inflammatory markers, age < 6 months, MAS features). Step 4: Initial therapy. Mild: IVIG monotherapy (2 g/kg). Moderate: IVIG + may be considered early UDCA (10–20 mg/kg/day). Glucocorticoid addition based on individual risk. Severe/High-risk: Preferred: initial combination therapy (IVIG + glucocorticoid initiated concurrently) + UDCA. Step 5: Response assessment (36-48h). Responder: Taper glucocorticoid; monitor bilirubin. Refractory: Escalate to anakinra (2–8 mg/kg/day) or tocilizumab. Plasma exchange reserved for bilirubin >170 μmol/L, coagulopathy, or suspected MAS. Clinical note: In severe cholestasis (bilirubin >85 μmol/L), delaying glucocorticoids pending IVIG response risks irreversible biliary epithelial injury. Initial combination therapy may be considered. Step 6: Follow-up. Weekly bilirubin/bile acids; echocardiography per AHA guidelines; hepatology follow-up if abnormalities persist >3 months. AHA, American Heart Association; CALs, coronary artery lesions; IVIG, intravenous immunoglobulin; MAS, macrophage activation syndrome; UDCA, ursodeoxycholic acid.

The ongoing ANACOMP Phase III trial (n=84, ClinicalTrials.gov: NCT04656184) comparing anakinra to second-dose IVIG in IVIG-resistant KD aims to provide robust evidence, but does not specifically stratify by cholestasis status (47). An integrated clinical algorithm incorporating the diagnostic, stratification, and therapeutic steps discussed above is outlined in **Figure 3**.

## Prognosis

### Short-term outcomes

Mild-to-moderate cholestasis typically resolves within 1–3 months with appropriate anti-inflammatory therapy (3). Poor short-term outcomes correlate with: (i) delayed diagnosis >10 days from onset; (ii) IVIG resistance requiring additional therapy; (iii) severe baseline cholestasis (direct bilirubin >85.5 μmol/L); and (iv) concomitant MAS. Severe cases have higher risk of transient coagulopathy and hypoalbuminemia requiring supportive care, but progression to acute liver failure is extremely rare (<0.1%).

### Long-term prognosis

Long-term follow-up studies indicate that KD-associated cholestasis resolves completely without progression to chronic liver disease, cirrhosis, or end-stage liver failure (3). This natural history contrasts with other cholestatic conditions (e.g., biliary atresia) and supports the reversible, inflammatory nature of KD hepatobiliary involvement.

However, the landmark 10- to 21-year follow-up study by Kato et al. demonstrated that coronary artery aneurysms develop in approximately 25% of untreated KD patients, with regression in 49% but persistent abnormalities in 31% and significant stenosis/occlusion in 20% (48). Given that cholestasis has been identified as an independent risk factor for coronary artery lesions (5, 6), regular echocardiographic surveillance is warranted in all patients with moderate-to-severe cholestasis regardless of biochemical resolution, with follow-up intervals per AHA guidelines based on CAL status.

## Unresolved controversies and knowledge gaps

### Fundamental mechanistic questions

Causality of the hepatic vascular-biliary unit hypothesis: Is peribiliary microvascular injury a primary cause or secondary consequence of cholestasis? Does toxic bile acid accumulation exacerbate microvascular injury, creating a feed-forward loop? Resolution requires conditional endothelial-specific knockout animal models with KD vasculitis phenotype.

Biomarker vs. mediator: Is cholestasis a passive biomarker of systemic inflammation severity, or an active mediator promoting CAL development through circulating bile acid-mediated vascular toxicity? This distinction has therapeutic implications—if cholestasis actively contributes to CAL pathogenesis, early and aggressive choleretic therapy (UDCA) might confer cardiovascular benefit independent of cholestasis resolution.

Bile acid toxicity to extrahepatic vasculature: Do elevated serum bile acids in KD contribute to coronary artery endothelial dysfunction or aneurysm formation, or is toxicity confined to the hepatobiliary system? *In vitro* studies of bile acid effects on coronary endothelial cells are needed.

Genetic susceptibility: Do known KD susceptibility variants (*ITPKC*, *CASP3*, *ORAI1*, *FCGR2A*) specifically predispose to hepatobiliary manifestations? Genotype-phenotype studies stratified by organ involvement are lacking.

### Clinical evidence gaps

Despite the progress made in characterizing the clinical features and pathogenic correlates of KD-associated cholestasis, substantial uncertainties remain regarding its underlying mechanisms, optimal management strategies, and long-term outcomes. To guide future research efforts and resource allocation, we have identified and prioritized the most pressing clinical evidence gaps that require urgent investigation through well-designed prospective studies; these are outlined in **Table 12**.

**Table 12:** Clinical evidence gaps.

Gap	Current status	Priority	Proposed study design
RCT comparing sequential vs. combination glucocorticoids in cholestasis subset	None	Highest	Multicenter RCT, n=200, stratified by cholestasis severity (moderate vs. severe), primary endpoint: time to bilirubin normalization; secondary: IVIG resistance, CAL development
RCT on optimal UDCA timing	None	High	Placebo-controlled RCT of UDCA initiated <48h vs. >7 days or post-IVIG response
Biologic therapy comparative effectiveness in cholestasis	Phase I/IIa trials, n<40	High	Prospective registry with cholestasis as stratification variable; comparative effectiveness of anakinra vs. tocilizumab
Long-term hepatobiliary outcomes	>10-year data lacking	Moderate	Prospective cohort with liver fibrosis assessment (elastography, enhanced liver fibrosis panel) at 5 and 10 years
Severity stratification validation	Proposed thresholds from single-center cohorts	Moderate	Prospective multicenter validation in East Asian, European, and North American populations
Diagnostic criteria validation	Expert consensus only	Moderate	Prospective diagnostic accuracy study against reference standard of expert panel adjudication
Cholestasis-MAS relationship	Case series only	Moderate	Nested case-control study within KD cohorts to define predictive biomarkers

### Atypical KD diagnostic dilemma

For infants presenting with isolated cholestasis as the sole manifestation of atypical KD, the optimal balance between early IVIG intervention (to prevent CALs) and avoiding overdiagnosis/treatment of alternative cholestatic conditions remains unresolved. Novel biomarkers (e.g., specific bile acid metabolomic signatures distinguishing KD from biliary atresia, genetic risk scores incorporating hepatobiliary-specific variants) are urgently needed to improve diagnostic accuracy in this high-risk, diagnostically challenging population (48, 49).

## Limitations

This review has several limitations that should be acknowledged.

First, this is a narrative review rather than a systematic review or meta-analysis. Although we conducted a comprehensive literature search across multiple databases (PubMed, Web of Science, Scopus, Embase) using keywords including “Kawasaki disease,” “cholestasis,” “hepatitis,” “hepatobiliary,” “hyperbilirubinemia,” and “liver dysfunction,” we did not apply formal systematic review methodology such as PRISMA guidelines, risk of bias assessment using Cochrane tools, or formal study quality scoring. Therefore, selection bias cannot be fully excluded, and our synthesis reflects qualitative rather than quantitative integration of evidence.

Second, the evidence base for the proposed hepatic vascular-biliary unit injury hypothesis is currently correlative rather than causal. The supporting evidence derives from human pathological specimens, immunohistochemistry, and *in vitro* experiments (OCEBM Level 3-4). Causality—whether peribiliary microvascular injury is proposed to trigger cholestasis or whether cholestasis exacerbates microvascular injury—remains unproven and requires validation in animal models with conditional endothelial-specific gene targeting and longitudinal mechanistic studies.

Third, the therapeutic recommendations for moderate-to-severe cholestasis (IVIG-glucocorticoid sequential vs. combination therapy) are based exclusively on retrospective cohort studies and case series (OCEBM Level 3-4), or extrapolated from RCTs in severe KD populations not specifically defined by cholestasis. While anakinra shows promise from Phase I/IIa trials, these studies have small sample sizes, lack control groups, and do not specifically report cholestasis outcomes. No randomized controlled trials have specifically addressed this subpopulation. Consequently, the therapeutic insights presented should be interpreted as hypothesis-generating and mechanistically rational rather than definitive evidence-based clinical guidance.

Fourth, we did not perform formal GRADE (Grading of Recommendations, Assessment, Development, and Evaluations) assessment for the proposed management strategies. The severity stratification thresholds (direct bilirubin 34.2 μmol/L and 85.5 μmol/L) are derived from single-center or regional cohorts and require prospective validation in diverse populations, particularly across Asian countries where KD incidence is highest and genetic background may influence disease phenotype.

Fifth, the relationship between cholestasis and other severe KD manifestations (particularly MAS and CNS involvement) is described based on pathophysiological reasoning and case observations rather than prospective studies defining predictive biomarkers or shared mechanistic pathways.

Despite these limitations, this review provides a comprehensive, critically appraised, and evidence-graded synthesis of the current knowledge on KD-associated cholestasis, establishes a novel pathogenic framework with explicit validation requirements, and delineates priority research directions to guide future investigation.

## Future research priorities

### Basic mechanistic research

Develop animal models recapitulating both KD vasculitis and cholestasis to validate the hepatic vascular-biliary unit hypothesis, specifically utilizing conditional endothelial-specific (VE-cadherin-Cre or Tie2-Cre) and cholangiocyte-specific (KRT19-Cre or Sox9-Cre) gene targeting to establish causality.

Define the causal relationship between microvascular injury and cholangiocyte dysfunction using time-course studies in these models, with serial assessment of bile acid transporter expression, serum biochemistry, and microvascular morphology.

Map the bile acid metabolomic landscape longitudinally from acute phase through convalescence using targeted and untargeted metabolomics, with correlation to inflammatory cytokine profiles and transporter expression.

Investigate genetic modifiers of hepatobiliary involvement through genome-wide association studies (GWAS) stratified by organ-specific phenotypes, and functional studies of identified variants in cholangiocyte and endothelial cell models.

### Clinical translational research

Conduct multicenter RCT comparing sequential vs. combination glucocorticoids in KD patients with confirmed cholestatic hepatitis (stratified by moderate vs. severe), with time to bilirubin normalization as primary endpoint and IVIG resistance, CAL development, and long-term liver fibrosis markers as secondary endpoints.

Evaluate early UDCA initiation in a placebo-controlled RCT specifically powered for cholestasis resolution and CAL prevention.

Establish a prospective international registry for biologic therapy in refractory cholestatic KD to generate comparative effectiveness data specifically for cholestasis outcomes, with standardized definitions and severity stratification.

Complete ongoing Phase III trials of anakinra (ANACOMP) and design subsequent trials with cholestasis as a predefined stratification variable and outcome measure.

Future multicenter studies across Asian countries—where KD incidence is highest—are needed to validate severity stratification thresholds, define population-specific genetic risk factors, and develop region-specific clinical algorithms incorporating local healthcare resource constraints.

### Diagnostic and prognostic biomarkers

Validate bile acid metabolomic panels as early diagnostic tools distinguishing KD-associated cholestasis from biliary atresia and viral hepatitis, particularly in atypical KD presentations.

Develop genetic susceptibility scores incorporating hepatobiliary-specific variants to identify high-risk patients at diagnosis for targeted intensified therapy.

Validate severity stratification thresholds in diverse populations and define optimal cutoffs for therapeutic decision-making.

Define predictive biomarkers for MAS development in patients with severe cholestasis to enable preemptive intervention.

### Standardization

Develop and prospectively validate unified international diagnostic criteria for KD-associated cholestasis, incorporating biochemical thresholds, imaging features, and exclusion criteria.

Establish standardized severity classification with validated thresholds and long-term hepatobiliary follow-up protocols, including non-invasive fibrosis assessment (elastography) for research purposes.

## Conclusion

KD-associated cholestatic hepatitis is a clinically significant extra-cardiac manifestation that serves as a critical sentinel of systemic hyperinflammation and an independent predictor of IVIG resistance and CALs. The hepatic vascular-biliary unit injury hypothesis provides an integrated pathogenic framework linking the core vasculitic process to cholangiocyte dysfunction and bile acid metabolism disturbances, though causality remains to be established through experimental validation.

Critically, the current evidence base for therapeutic decision-making in moderate-to-severe cholestasis is weak. Most recommendations derive from retrospective cohorts, case series, or extrapolation from severe KD trials not specific to cholestasis (OCEBM Level 3-4). No RCT has specifically addressed optimal glucocorticoid regimen selection in this subpopulation. Emerging evidence supports interleukin-1 blockade with anakinra for refractory cases, but data remain limited to Phase I/IIa trials and case series predominantly focused on coronary artery outcomes rather than cholestasis-specific efficacy.

This review delineates these evidence gaps, proposes specific validation requirements for the hepatic vascular-biliary unit hypothesis, and establishes priority research directions. High-quality RCTs specifically targeting the cholestasis subpopulation and mechanistic studies employing conditional genetic models are urgently needed to transform the management of KD-associated cholestasis from expert opinion to evidence-based practice. Given the elevated incidence and potential for severe outcomes in East Asian populations, clinicians in this region should maintain particular vigilance for this under-recognized manifestation of systemic vasculitis. Meanwhile, these thresholds have not been prospectively validated and should be used solely for future research design, not for clinical decision-making.
